# Revision of the genus *Ceriantheomorphe* (Cnidaria, Anthozoa, Ceriantharia) with description of a new species from the Gulf of Mexico and northwestern Atlantic

**DOI:** 10.3897/zookeys.874.35835

**Published:** 2019-09-09

**Authors:** Celine S.S. Lopes, Hellen Ceriello, André C. Morandini, Sérgio N. Stampar

**Affiliations:** 1 Universidade Estadual Paulista (UNESP), Departamento de Ciências Biológicas, Laboratório de Evolução e Diversidade Aquática – LEDA/FCL, Avenida Dom Antônio, 2100 – Parque Universitário, Assis, São Paulo, Brazil Universidade Estadual Paulista Botucatu Brazil; 2 Universidade Estadual Paulista (UNESP), Instituto de Biociências, Departamento de Zoologia, Rua Prof. Dr. Antônio Celso Wagner Zanin, 250 – Distrito de Rubião Junior, Botucatu, São Paulo, Brazil Universidade de São Paulo São Paulo Brazil; 3 Universidade de São Paulo (USP), Instituto de Biociências – Departamento de Zoologia, Rua do Matão, Travessa 14, 101, Cidade Universitária, São Paulo, Brazil Universidade Estadual Paulista Botucatu Brazil; 4 Universidade de São Paulo (USP), Centro de Biologia Marinha (CEBIMar), Rodovia Manoel Hypólito do Rego, Km 131.50, Praia do Cabelo Gordo, São Sebastião, São Paulo, Brazil Universidade de São Paulo São Paulo Brazil

**Keywords:** Biogeography, cnidarian taxonomy, North America, Pacific Ocean, South America

## Abstract

The present study presents a revision of the genus *Ceriantheomorphe* Carlgren, 1931, including redescriptions of the two presently recognized species, *Ceriantheomorphe ambonensis* (Kwietniewski, 1898) and *Ceriantheomorphe brasiliensis* (Mello-Leitão, 1919), **comb. nov.**, and a description of the new species *Ceriantheomorphe adelita***sp. nov.**

## Introduction

Ceriantharia is a subclass within the cnidarian class Anthozoa, consisting of species commonly known as tube anemones. This taxon has several taxonomic inconsistencies (Stampar et al. 2016) that are understudied, in part because of sampling difficulties (den Hartog 1977; Stampar et al. 2016). Moreover, ecology, behavior and life cycle in most species are poorly known (Nyholm 1943; Stampar et al. 2015, 2016). Also, most systematic studies are solely based on morphological characters of few specimens (Carlgren 1912; Arai 1965; den Hartog 1977), leading to unreliable terminology (Arai 1965), and contributing to taxonomic uncertainty. A combination of these problems occurs in the genus *Ceriantheomorphe* Carlgren, 1931, which currently includes only two species: *Ceriantheomorphe brasiliensis* sensu Carlgren, 1931, and *Ceriantheomorphe ambonensis* (Kwietniewski 1898) (Carlgren 1931; den Hartog 1977).

The genus *Ceriantheomorphe* was described by Carlgren (1931) through the description of *C. brasiliensis* from southeastern Brazil. In this study, Carlgren also proposed that two species, *Cerianthus ambonensis* Kwietniewski, 1898 described from Ambon, Indonesia and *Cerianthus brasiliensis* Mello-Leitão, 1919 described from Guanabara Bay (Rio de Janeiro, Brazil) should be reassigned to the genus *Ceriantheomorphe*. As well, Carlgren (1931) also pointed out that *Cerianthus brasiliensis* is likely a synonym of *Ceriantheomorphe brasiliensis* sensu Carlgren 1931.

However, assigning *Cerianthus ambonensis* as “*Ceriantheomorphe ambonensis*” would have been premature because the simple description made by Kwietniewski (1898) did not include any mention of deposited type material. Additionally, Carlgren (1931) was not able to observe the holotype of *Cerianthus brasiliensis* described by Mello-Leitão (1919), so his assignment of the species to *Ceriantheomorphe* must be viewed as tentative.

More than two decades after the description of *Ceriantheomorphe*, Carlgren and Hedgpeth (1952) reported *C. brasiliensis* from the Gulf of Mexico. However, the authors suggested that these specimens could possibly be another species due to the disjunct occurrence in relation to South American specimens (Carlgren and Hedgpeth 1952; den Hartog 1977; Spier et al. 2012).

Despite this taxonomic confusion, *Ceriantheomorphe brasiliensis* had been listed as an endangered species in Brazil for over 10 years (MMA 2004). Furthermore, the tubes built by *C. brasiliensis*, and Ceriantharia in general, play an important ecological role in providing suitable alternative substrates to many invertebrate groups (e.g., Bryozoa, Crustacea, Anthozoa) (Tiffon 1987; Kim and Huys 2012; Vieira and Stampar 2014). For example, some species, such as the phoronid *Phoronis australis* Haswell, 1883, are only found in ceriantharian tubes (Stampar et al. 2010). Thus, the survival of *P. australis* may be related to the management of the cerianthid species that houses them in the southern Atlantic.

This study aims to present a taxonomic review of the genus *Ceriantheomorphe* including a redescription of the holotype of *C. brasiliensis*, a redescription of *C. ambonensis*, and the description of a new species from the North Atlantic.

## Material and methods

### Specimens

Twenty specimens of *Ceriantheomorphe* were sampled by SCUBA: sixteen of *C. brasiliensis* from the South Atlantic, three from the North Atlantic, and one, *C. ambonensis*, from the Pacific Ocean (Table 1).

**Table 1.** List of *Ceriantheomorphe* specimens in this study. Abbreviations: ES = Espírito Santo State; RJ = Rio de Janeiro State; SP = São Paulo State; SC = Santa Catarina State; UFRJ Biologia = cnidarian collections of the Department of Zoology, Biology Institute, Universidade Federal do Rio de Janeiro, Brazil; MZSP = Zoology Museum, Universidade de São Paulo, Brazil; MNHN Montevideo = National Museum of Natural History, Montevideo, Uruguay; USNM = United States National Museum, Washington DC, USA.

Species	Country	Locality	Coordinates	Museum code
*C. brasiliensis*	Brazil	Guanabara Bay-RJ	22°49'6''S, 43°8'45''W	MNRJ 200
		Arraial do Cabo-RJ	23°0'4''S, 42°0'29''W	MZSP 8470
		Araçá Beach-SP	23°48'58''S, 45°24'24''W	MZSP 8471
		Araçá Beach-SP	23°48'58''S, 45°24'24''W	MZSP 8472
		Cagarras Islands-RJ	23°1'55''S, 43°11'58''W	MZSP 8473
		Canasvieiras-SC	27°25'31''S, 48°27'0.2''W	MZSP 8475
		Camburi Beach-ES	20°16'39''S, 40°16'29''W	UFRJ Biologia 0293
		Camburi Beach-ES	20°16'39''S, 40°16'29''W	UFRJ Biologia 0337
		Rio de Janeiro-RJ	–	UFRJ Biologia 2-141
		Urca-RJ	–	UFRJ Biologia 2-086
		Zimbro Beach-SP	23°49'27''S, 45°25'4''W	UFRJ Biologia 2-11
		Sabacu Island-RJ	23°0'43''S, 44°22'7''W	MNRJ 2766
	Uruguay	José Ignacio-Maldonado	35°00'S, 54°24'2''W	MZSP 8474
		La Paloma-Rocha	34°42'3''S, 54°0.5'W	UFRJ-Biologia 2-464 A
		La Paloma-Rocha	34°42'3''S, 54°0.5'W	UFRJ-Biologia 2-464 B
		Punta del Diabo	34°04'S, 53°29'W	MNHN Montevideo I-1168
*C. adelita* sp. nov.	Mexico	Punta de Almagre-Tamaulipas	–	USNM 50016
	United States of America	Pass A’Loutre-Louisiana	–	USNM 51253
		Port Aransas, Corpus Christi-Texas	–	USNM 50015
*C. ambonensis*	Indonesia	Jakarta Bay-Jakarta	–	MZSP 8476

### Morphological studies

The morphology of all specimens was studied through internal anatomy and cnidome studies, both based on criteria adopted by Carlgren (1931), Arai (1965), den Hartog (1977) and Stampar et al. (2015). All specimens were observed separately. Specimens were longitudinally dissected along the ventral side using surgical scalpels, photographed under an Opticam stereomicroscope, using the OPT HD 3.7 software and a general description of each body region was made. The morphological characters were compared between specimens and descriptions available in the relevant literature (Kwietniewski 1898; Carlgren 1931; Spier et al. 2012).

All protomesenteries/directive mesenteries (P) were measured. Five quartets of mesenteries were measured for each specimen. We also divided the metamesenteries (type M and type m) value and betamesenteries (type B and type b) value to calculate the ratio between these mesentery types. We calculated the proportion occupied by protomesenteries in the gastrovascular cavity using the following equation:

F (length of protomesentery) × 100 / E (length of gastrovascular cavity)

The cnidome study was based on the sampling of 30 cnidae capsules for each cnida type from each body region (superior tip of marginal and labial tentacles, actinopharynx region, column, metamesenteries and betamesenteries). Each cnida was classified according to their shape based on different authors (Mariscal 1974; den Hartog 1977; Stampar et al. 2015) and measured using a Nikon Eclipse E200 microscope and MOTIC IMAGES PLUS 2.0 imaging software.

## Systematic results

### Phylum Cnidaria Verrill, 1865

#### Class Anthozoa Ehrenberg, 1834

**Subclass Ceriantharia Perrier, 1893**

**Suborder Spirularia den Hartog, 1977**

**Family Cerianthidae Milne-Edwards & Haime, 1851**

##### Genus Ceriantheomorphe

Taxon classificationAnimaliaSpirulariaCerianthidae

Carlgren, 1931

06A73769A3A05B46892CA1387B2900BE

###### Diagnosis.

Cerianthidae with fertile mesenteries, except for directives. Two pairs of mesenteries connected to the siphonoglyph. Mesenteries grouped in quartets following M, B, m, b order (after Carlgren 1931; Spier et al. 2012).

###### Type species.

*Ceriantheomorphe brasiliensis* (Mello-Leitão, 1919).

###### Valid species.

*Ceriantheomorphe brasiliensis* (Mello-Leitão, 1919) new comb., *Ceriantheomorphe ambonensis* (Kwietniewski, 1898), *Ceriantheomorphe adelita* sp. nov.

###### Distribution.

Southwestern Atlantic (Brazil and Uruguay), Gulf of Mexico (United States of America and Mexico), Central West Pacific (Java Sea, Indonesia).

##### Ceriantheomorphe brasiliensis

Taxon classificationAnimaliaSpirulariaCerianthidae

(Mello-Leitão, 1919) comb. nov.

D2A61343A5855194A6177055A4AA4330

Fig. 1 A–C

Cerianthus brasiliensis Mello-Leitão, 1919: 38–39.
Ceriantheomorphe brasiliensis sensu Carlgren 1931: 2–6; Carlgren 1940: 6, 11–12; Carlgren and Hedgpeth 1952: 148, 169–170; Frey 1970: 309; Molodtsova 2009: 365–367; Stampar et al. 2010: 205–209; Silveira and Morandini 2011: 3; Rodriguez et al. 2011: 52, 54–55; Spier et al. 2012: 1–3; Stampar et al. 2012: 5–6, 9; Stampar et al. 2014a: 2, 5, 8; Stampar et al. 2014b: 344, 347, 351, 353; Stampar and Morandini 2014: 2; Vieira and Stampar 2014: 370; Stampar et al. 2015: 3; González-Muñoz et al. 2016: 5, 9; Stampar et al. 2016: 64, 67, 68.
Ceriantheomorphe brasiliensis (not) – Hedgpeth 1954: 286.

###### Material examined (16 specimens).

**Figure 1.**
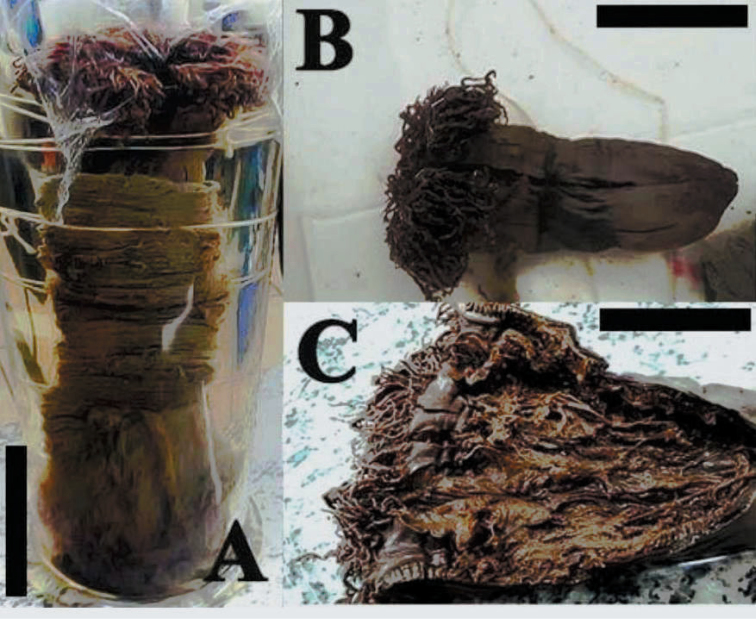
Holotype of *Ceriantheomorphe brasiliensis* (MNRJ 200). **A** Specimen inside the tube **B** specimen without the tube **C** dissected specimen. Scale bars: 2 cm.

**Figure 2.**
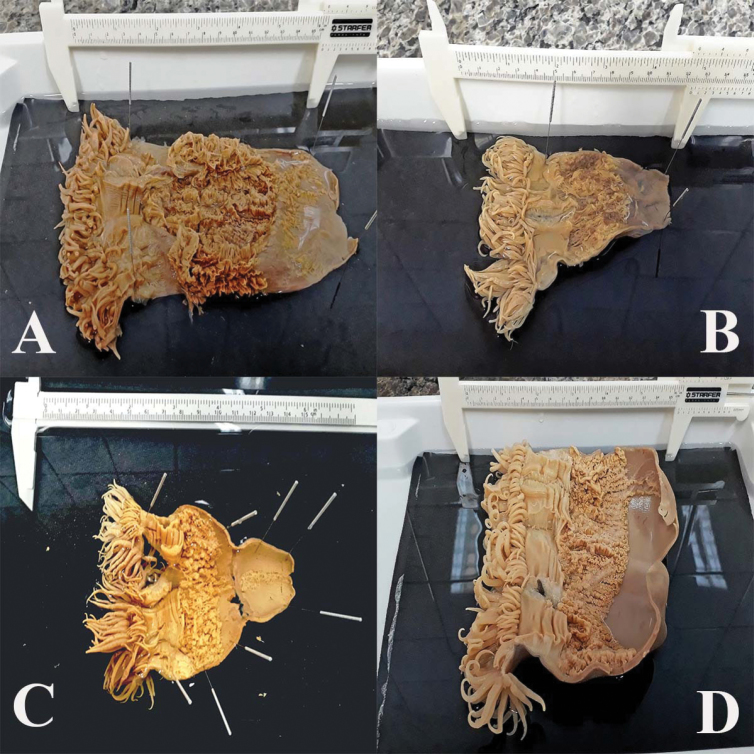
Dissected specimens of *Ceriantheomorphe brasiliensis* from southwestern Atlantic. **A** Individual UFRJ Biologia 0293 from Camburi (ES) **B** specimen UFRJ Biologia 0337 from Camburi (ES) **C** specimen MNHN Montevideo I-1168 from Punta del Este (Uruguay) **D** individual from Canasvieiras Beach, Santa Catarina.

**Holotype**: MNRJ 200 • adult individual (16.5 cm long), Guanabara Bay, Rio de Janeiro, Brazil (22°49'6"S, 43°8'45"W), Mello-Leitão leg. (Fig. 1 A–C). **Paratypes**: MZSP 8470 • adult individual (9.3 cm long), Arraial do Cabo (near Farol Island, 18 m depth), Rio de Janeiro, Brazil (23°0'4"S, 42°0'29"W), S.N. Stampar leg. (20/i/2009); MZSP 8471 • adult individual (24 cm long), Araçá Beach (intertidal), São Sebastião, São Paulo, Brazil (23°48'58"S, 45°24'24"W), J.A. Petersen leg. (03/ii/1965); MZSP 8472 • adult individual (16.5 cm long), same locality data as for preceding; MZSP 8473 • juvenile individual, (8.5 cm long), Cagarras Islands (22 m depth), Rio de Janeiro, Brazil (23°1'55"S, 43°11'58"W), S.N. Stampar leg. (15/iv/2009); MZSP 8474 • adult individual (22.2 cm long), José Ignacio (27 km from the coast, 38 m depth), Maldonado, Uruguay (35°00'S, 54°24'2"W), F. Scarabino leg. (18/ix/2005); MZSP 8475 • adult individual (14.4 cm long), Canasvieiras Beach, Florianópolis (4 m depth), Santa Catarina, Brazil, (27°25'31"S, 48°27'0.2"W), S.N. Stampar leg. (21/i/2009) (Fig. 2D); UFRJ Biologia 0293 • adult individual (17 cm long), Camburi Beach, Espírito Santo, Brazil (20°16'39"S, 40°16'29"W), (18/viii/1989) (Fig. 2A); UFRJ Biologia 0337 • adult individual (16.5 cm long), same data as for preceding, (17/iv/1990) (Fig. 2B); UFRJ Biologia 2-141 • adult individual (22 cm long), Rio de Janeiro, Brazil, A. Saldanha leg. (1966); UFRJ Biologia 2-086 • damaged adult individual, Urca, Rio de Janeiro, Brazil, (1959); UFRJ Biologia 2-11 • adult individual (10.9 cm long), Zimbro Beach, São Sebastião (4–6 m depth), São Paulo, Brazil, (23°49'27"S, 45°25'4"W), E.Q. Cez leg. (04/ix/1967); UFRJ Biologia 2-464 A • damaged individual, (34 m depth), La Paloma, Uruguay, (34°42'3''S, 54°0.5'W), Conversut I #4557 exped. (17/ix/77); UFRJ Biologia 2-464 • damaged individual, same data as for preceding specimen; MNRJ 2766 B • adult individual (14.5 cm long), Sabacu Island, Angra dos Reis (6 m depth), Rio de Janeiro, Brazil (23°0'43"S, 44°22'7"W), C.C. Ratto leg. (07/xii/1993); MNHN Montevideo I-1168 • adult individual (11 cm long), Rocha (6 km from the coast, in line of Santa Teresa Fortress, 18 m depth), Punta del Diabo, Uruguay (34°04'S, 53°29'W), Navio Hero (3A) exped. (21/vii/1972) (Fig. 2C).

###### Diagnosis.

**Figure 3.**
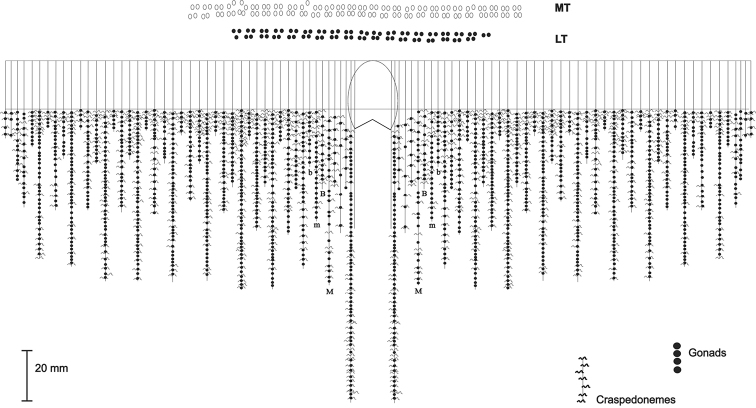
Mesenteries arrangement of the holotype of *Ceriantheomorphe brasiliensis* (MNRJ 200). **MT** Marginal tentacles **LT** Labial tentacles **M** and **m** Metamesenteries **B** and **b** Betamesenteries.

Large cerianthid, 8.5–24 cm long and 1.5–13.8 cm wide. 132–392 marginal tentacles arranged in (1)1123.1123 and 108–384 labial tentacles arranged in (1)1122.1122 or (1)1123.1123. Pharynx occupies about 8–27% of total body length. Five pairs of protomesenteries, of which two pairs connected to the siphonoglyph, (directives and P2). Gastrovascular cavity takes up to 33–72% of total body length. All fertile mesenteries, except for directives. Number of mesenteries about 170–642. Directives mesenteries longer than protomesenteries P3, P5 and metamesenteries m, except by m of the 2^nd^ and 3^rd^ cycles. Protomesenteries (P2) longer than all mesenteries, extending up to the aboral pore (Fig. 3). Protomesenteries (P3) shorter than protomesenteries (P4) and longer than protomesenteries (P5) and betamesenteries (B), except by B of the 1^st^ and 2^nd^ cycles. Protomesenteries (P4) longer than directive mesenteries, P5 and metamesenteries (m), except by m of the 2^nd^ and 3^rd^ cycles. Protomesenteries (P5) shorter than all others protomesenteries and metamesenteries M and m. Ratio of 1.2–3.1% between betamesenteries (B × b) and 1.1–3.1% between metamesenteries (M × m). Directive mesenteries, protomesenteries P3, P4 and P5 occupies about 36.6%, 12.2%, 38.8% and 11.1% of total gastrovascular cavity length, respectively, while protomesenteries P2 extend over 80%. Cnidome composed of spirocysts, microbasic b-mastigophores (six types), atrichous (two types), ptychocysts and holotrichous (Fig. 5A–J, Table 2).

**Table 2.** Measurements of 30 cnida capsules for each cnida type in 6 distinct body regions of *Ceriantheomorphe brasiliensis* (N = 16). Information inside parentheses indicates cnidae length and width, respectively, and information outside parentheses indicates average of cnidae size.

Body part/cnida type	*Ceriantheomorphe brasiliensis*
**Marginal tentacles**	
Microbasic b-mastigophore type I	65.56 (50.50–80.63) × 13.13 (7.57–18.69)
Microbasic b-mastigophore type II	38.23 (27.96–48.5) × 4.99 (3.13–6.86)
Microbasic b-mastigophore type III	31.16 (18.36–43.97) × 3.96 (1.97–5.95)
Microbasic b-mastigophore type IV	16.55 (10.61–22.49) × 4.10 (2.2–6.01)
Microbasic b-mastigophore type V	27.87 (18.01–37.73) × 7.02 (1.6–5.42)
**Labial tentacles**	
Microbasic b-mastigophore type I	48.75 (36.89–60.61) × 9.11 (5.41–12.82)
Microbasic b-mastigophore type II	34.93 (25.2–44.66) × 5.12 (3.65–6.6)
Microbasic b-mastigophore type III	28.27 (17.20–39.35) × 4.03 (1.71–6.35)
Microbasic b-mastigophore type IV	24.11 (17.25–30.97) × 2.73 (1.64–3.83)
Microbasic b-mastigophore type V	26.10 (15.03–37.18) × 3.29 (1.79–4.79)
**Pharynx**	
Atrichous type I	38.33 (26.15–50.52) × 5.95 (2.68–9.22)
Microbasic b-mastigophore type I	52.64 (35.56–69.73) × 8.38 (5.43–11.33)
Microbasic b-mastigophore type II	44.39 (32.10–56.68) × 6.09 (3.28–8.91)
Microbasic b-mastigophore type III	34.97 (21.86–48.09) × 3.35 (2.13–4.57)
Microbasic b-mastigophore type V	27.62 (23.37–31.88) × 2.81 (2.19–3.43)
**Column**	
Ptychocyst type I	71.99 (56.21–87.77) × 24.41 (13.75–35.08)
Ptychocyst type II	77.14 (50.15–94.14) × 14.12 (8.86–19.38)
Atrichous type I	48.85 (30.09–67.61) × 11.09 (4.41–17.78)
Microbasic b-mastigophore type I	41.33 (26.47–56.2) × 6.25 (3.96–8.54)
Microbasic b-mastigophore type IV	28.14 (23.83–32.45) × 3.12 (2.48–3.76)
Microbasic b-mastigophore type V	29.95 (22.51–37.4) × 3.03 (2.08–3.98)
Holotrichous	50.95 (30.04–71.86) × 14.88 (7.3–22.46)
**Mesenteries M**	
Microbasic b-mastigophore type I	51.58 (35.0–68.17) × 10.09 (6.41–13.77)
Microbasic b-mastigophore type IV	22.25 (10.93–33.58) × 5.76 (2.25–9.28)
Microbasic b-mastigophore type III	20.03 (13.3–26.77) × 4.90 (2.91–6.9)
**Mesenteries b**	
Microbasic b-mastigophore type I	54.65 (39.57–69.74) × 10.44 (7.16–13.73)
Microbasic b-mastigophore type II	33.69 (24.83–42.56) × 5.01 (3.32–6.7)
Microbasic b-mastigophore type III	19.97 (12.1–27.85) × 4.17 (1.95–6.4)
Microbasic b-mastigophore type IV	19.59 (8.62–30.56) × 4.06 (2.24–5.89)

###### Distribution.

Southwestern Atlantic-Brazil (from the State of Espírito Santo (20.5°S) to Rio Grande do Sul (33.7°S) State) and Uruguay (34°S). This species was only observed in shallow waters (1–40 m depth).

###### Description of holotype.

(MNRJ 200) (Fig. 1A–C). Large ceriantharian, 16.5 cm long and 7.7–10.4 cm wide. 388 marginal tentacles (4.9 cm long in preserved specimen) and 312 labial tentacles (1.7 cm long in preserved specimen), both disposed in four cycles. Marginal tentacles arrangement: (1)1243.1243.1123.1123…, labial tentacles arrangement: (1)1234.1122.1243.1243… Small pharynx, 15% of total body length, well-marked siphonoglyph. Five pairs of protomesenteries, two of which connected to the siphonoglyph. Indistinct hyposulcus and hemisulci. With exception of short directives, all mesenteries are fertile. Long protomesenteries P2 extending up to the aboral pore and longer than metamesenteries all mesenteries. Arrangement of mesenteries is M,B,m,b (Fig. 3). Mesenteric filaments of almost the same length of mesenteries. Craspedonemes only on initial part of gastrovascular cavity. Cnidome composed of spirocysts, microbasic b-mastigophores (two types), atrichous and ptychocyst.

##### Ceriantheomorphe ambonensis

Taxon classificationAnimaliaSpirulariaCerianthidae

E74D3389DABF53709E1994C01693FE34

Fig. 6A–B

Cerianthus ambonensis Kwietniewski, 1898: 426; Pax 1910: 167; McMurrich 1910: 26–28; Carlgren 1912: 44–47.
Cerianthus sulcatus McMurrich, 1910: 28–30.
Ceriantheomorphe ambonensis – Carlgren 1931: 1.

###### Material examined.

**(MZSP 8476)**: • young individual (3.8 cm long) from Jakarta Bay, Indonesia, K. Cassiolato leg. (viii/2011) (Fig. 6A–B).

###### Diagnosis.

Small cerianthid, 3.8 cm long and 2.1 cm wide. 48 marginal tentacles and 72 labial tentacles, both disposed in three cycles. Directive marginal and labial tentacles absent. Marginal tentacles arrangement: (0)1123.1121.1213.1213... Labial tentacles arrangement: (0)112.1121.1121.1121… Pharynx occupies about 18% of total body length. Hyposulcus and hemisulci absent. Gastrovascular cavity occupies about 55% of total body length. Three pairs of protomesenteries, all connected to the siphonoglyph (directive mesenteries, P2 and P3). About 96 mesenteries arranged in M,B,m,b (Fig. 7). Directive mesenteries shorter than all other mesenteries. Protomesenteries (P2) longer than all metamesenteries. Ratio of 4% between betamesenteries (B × b) and 2.2–3.5% between metamesenteries (M × m). Directive mesenteries, protomesenteries P2 and P3, occupy 2.3%, 85.7%, 14.2% of total gastrovascular cavity length, respectively. Cnidome (Fig. 8, Table 3) composed of spirocysts, microbasic b-mastigophores (six types), atrichous (one type), ptychocyst and holotrichous.

**Table 3.** Measurements of 30 cnida capsules for each cnida type in 6 distinct body regions of *Ceriantheomorphe ambonensis* (N = 1). Information inside parentheses indicates cnidae length and width, respectively, and information outside parentheses indicates average of cnidae size.

Body part/cnida type	*Ceriantheomorphe ambonensis*
**Marginal tentacles**	
Microbasic b-mastigophore type II	36.02 (23.16–48.89) × 6.18 (4.89–7.47)
Microbasic b-mastigophore type IV	19.54 (14.42–24.66) × 6.18 (4.89–7.47)
Microbasic b-mastigophore type V	18.90 (16.21–21.60) × 2.56 (2.22–2.90)
**Labial tentacles**	
Microbasic b-mastigophore type I	46.84 (42.40–51.28) × 8.05 (6.46–9.65)
Microbasic b-mastigophore type II	30.31 (26.15–34.47) × 4.58 (3.30–5.87)
Microbasic b-mastigophore type III	27.68 (24.16–31.20) × 3.54 (2.83–4.25)
Microbasic b-mastigophore type V	23.52 (18.13–28.92) × 2.82 (2.05–3.59)
**Pharynx**	
Atrichous	40.36 (33.48–47.25) × 5.99 (4.81–7.17)
Microbasic b-mastigophore type I	50.45 (44.63–56.28) × 7.49 (5.92–9.07)
Microbasic b-mastigophore type II	36.49 (32.28–40.70) × 5.17 (3.58–6.76)
Microbasic b-mastigophore type III	29.92 (24.42–35.42) × 3.59 (2.48–4.71)
**Column**	
Ptychocyst	61.96 (53.31–70.62) × 21.63 (17.22–26.05)
Atrichous	48.50 (41.69–55.32) × 11.38 (8.74–14.03)
Microbasic b-mastigophore type I	41.45 (34.51–48.39) × 9.64 (8.74–10.54)
Holotrichous	55.10 (47.45–62.76) × 14.97 (11.27–18.68)
**Mesenteries M**	
Microbasic b-mastigophore type I	49.11 (43.91–54.31) × 9.24 (6.92–11.57)
Microbasic b-mastigophore type IV	19.03 (16.70–21.37) × 4.99 (3.38–6.61)
**Mesenteries b**	
Microbasic b-mastigophore type IV	22.34 (16.34–28.34) × 5.93 (4.10–7.76)

###### Distribution.

Indonesia, shallow waters.

###### Description of specimen.

Small individual, with 3.8 cm long and 2.1 cm wide. 48 marginal tentacles and 72 labial tentacles, both disposed in three cycles. Marginal tentacles arrangement: (0)1123.112…, labial tentacles arrangement: (0)112.112.112… Small pharynx, occupies 18% of total body length. Hyposulcus and hemisulci absent. Well-marked siphonoglyph with three pairs of mesenteries connected to it (one pair of directive mesenteries and two pairs of protomesenteries). Long protomesenteries (P2) extending to the terminal pore and longer than other mesenteries. Directive mesenteries shorter than all mesenteries. Protomesenteries (P3) shorter than metamesenteries (M and m) and longer than betamesenteries (B and b). 96 mesenteries arranged in M,B,m,b (Fig. 6). Mesenteric filaments and craspedonemes present on initial portion of the gastrovascular cavity. Gastrovascular cavity occupies approximately 55% of the entire body length. Directive mesenteries and protomesenteries P3 occupy 2.3% and 14.2% of total gastrovascular cavity length, respectively, while protomesenteries P2 occupies 85.7%. Ratio of 2.2–3.5% between metamesenteries (M × m) and 4% between betamesenteries (B × b). Cnidome (Fig. 7) composed of spirocysts, microbasic b- mastigophores (six types), atrichous (one type), ptychocyst and holotrichous.

##### Ceriantheomorphe adelita

Taxon classificationAnimaliaSpirulariaCerianthidae

Lopes, Morandini & Stampar sp. nov.

27D64B3704735895842BF9F5226B24C4

http://zoobank.org/702BDFDD-870C-43EB-B59A-05A994177D56

Fig. 9A–B

Ceriantheomorphe brasiliensis Carlgren, 1931 (in part): 2–6; Carlgren and Hedgpeth 1952: 148, 169–170; Hedgpeth 1954: 286–290; Molodtsova 2009: 365–367; Stampar et al. 2010: 205–209; Spier et al. 2012: 1–3.

###### Material examined (3 specimens).

**Holotype**: USNM 50015 • adult individual, 19 cm long and 5.4–7.3 cm wide, Port Aransas, 32 km South off Corpus Christi, Texas, United States of America, W. Close leg. 07/ix/1947 (Fig. 9B). **Paratypes**: USNM 50016 • damaged individual, Tamaulipas, Punta de Almagre to North of Hut’s Bayo, Pelican R/V exped. 17/iii/1949; USNM 51253 • damaged juvenile individual, 5.0–5.9 cm wide from Pass A’Loutre (22 m depth), Louisiana, United States of America, Oh Johnny R/V exped. 25/vi/1969 (Fig. 9A).

###### Diagnosis.

Large cerianthid, 19 cm long and 5.0–7.3 cm wide. 192–352 marginal tentacles (2.4–3.0 cm long in preserved animal) and 144 to 336 labial tentacles (0.5–2.0 cm long in preserved animal), both disposed in four cycles. Marginal tentacles arrangement: (0)1123.1122.1122.1123.1122…, labial tentacles arrangement: (0)1123. 1122… Siphonoglyph well-marked by two protuberant tissues. Three pairs of protomesenteries (directive mesenteries, P2 and P3), all connected to the siphonoglyph. Well distinct hyposulcus and hemisulci absent. Protomesenteries (P3) longer than metamesenteries (m). Ratio from 2.7–5.2% between metamesenteries (M × m) and 3% between betamesenteries (B × b). Directive mesenteries, P2 and P3, extend up to 30.5%, 92.5% and 56.4% of total gastrovascular cavity length, respectively. Cnidome (Fig. 10A–I, Table 4) composed of spirocysts, microbasic b-mastigophores (five types), atrichous (two types) and ptychocyst.

**Table 4.** Measurements of 30 cnida capsules for each cnida type in 6 distinct body regions of *Ceriantheomorphe adelita* sp. nov. (N = 3). Information inside parentheses indicates cnidae length and width, respectively, and information outside parentheses indicates average of cnidae size.

Body part/cnida type	*Ceriantheomorphe adelita* sp. nov.
**Marginal tentacles**	
Microbasic b-mastigophores II	39.19 (34.20–44.18) × 5.04 (4.07–6.01)
Microbasic b-mastigophores V	25.12 (20.16–30.09) × 3.04 (2.09–3.99)
**Labial tentacles**	
Microbasic b-mastigophores I	48.72 (39.22–58.22) × 6.71 (5.24–8.19)
Microbasic b-mastigophores II	36.32 (28.18–44.46) × 4.77 (3.55–6.00)
**Pharynx**	
Atrichous	41.66 (32.23–51.09) × 5.74 (4.13–7.35)
Microbasic b-mastigophores I	51.43 (40.10–62.77) × 7.7 (6.25–9.15)
Microbasic b-mastigophores II	44.2 (35.29–53.11) × 5.13 (3.97–6.29)
Microbasic b-mastigophores III	36.75 (27.57–45.93) × 3.36 (2.53–4.20)
**Column**	
Atrichous	48.12 (38.88–57.37) × 9.28 (7.38–11.19)
Ptychocysts type I	55.42 (50.08–60.77) × 13.92 (9.49–18.35)
Microbasic b-mastigophores I	41.74 (47.80–35.68) × 6,0 (4.16–7.84)
Ptychocysts type II	64.3 (58.8–69.8) × 17.1 (15.0–19.2)
**Mesenteries M**	
Microbasic b-mastigophores IV	18.77 (23.27–14.27) × 4.24 (2.5–5.99)
**Mesenteries b**	
Microbasic b-mastigophores II	38.76 (34.01–43.51) × 4.36 (3.53–5.20)
Microbasic b-mastigophores III	19.94 (15.46–24.42) × 4.79 (3.98–5.60)
Microbasic b-mastigophores IV	23.37 (19.45–27.29) × 3.12 (2.15–4.10)

###### Etymology.

The specific name “*adelita*” is an allusion to an important group of women that fought during the Mexican Revolution. Occasionally, they adopted the identities of men to join in combat against the enemy.

###### Distribution.

Gulf of Mexico (Northern Mexico) to North Atlantic (North Carolina, United States of America), shallow waters.

###### Description of holotype.

USNM 50015, adult specimen, 19 cm long and 5.4–7.3 cm wide. 352 marginal tentacles (2.7 cm long in preserved animal) and 336 labial tentacles (2.0 cm long in preserved animal), both disposed in four cycles. Marginal tentacles arrangement: (0)1132.1122.1123.1122.1122…, labial tentacles arrangement (0)1122.1122… Directive tentacle absent. Pharynx occupies about 21% of entire body length, siphonoglyph well-marked by two lateral protuberances. Three pairs of mesenteries connected to the siphonoglyph. Gastrovascular cavity taking up to 56% of total body length. Mesenteric filaments of almost the same length of mesenteries; with craspedonemes only in the initial part of the gastrovascular cavity. Distinct hyposulcus and hemisulci absent. Fertile mesenteries, except for the directives. About 236 mesenteries arranged in M,B,m,b (Fig. 11). Directive mesenteries longer than betamesenteries (b and B) and metamesenteries (m). Protomesenteries (P2) extend to aboral pore. Protomesenteries (P3) longer than directive mesenteries, betamesenteries (B and b) and metamesenteries (m). Ratio from 2.7–5.2% between metamesenteries (M × m) and 3% between betamesenteries (B × b). Directive mesenteries and P3 extend up to 30.5%, and 56.4% of total gastrovascular cavity length, respectively, while protomesenteries (P2) occupy 92.5%. Cnidome composed of microbasic b-mastigophores (five types), atrichous (two types) and ptychocyst (Fig. 10A–I, Table 4).

### Comparison between congeners

**Figure 4.**
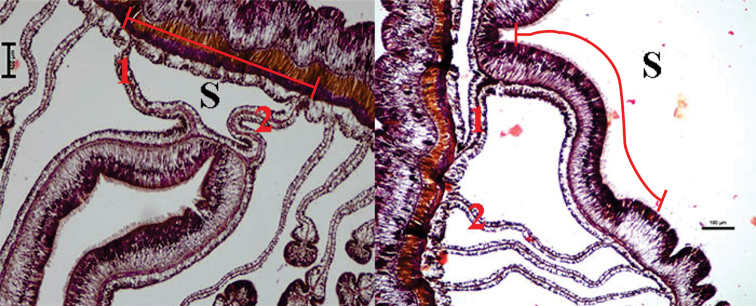
*Ceriantheomorphe brasiliensis* sectioned at actinopharynx region, showing mesenteries connected to the siphonoglyph. **S** Siphonoglyph area, 1 and 2. Mesenteries connected to the siphonoglyph.

Both *Ceriantheomorphe brasiliensis* and *C. adelita* sp. nov. have labial and marginal tentacles disposed in four cycles, whereas *C. ambonensis* has its tentacles arranged in three cycles. All three species have distinct labial and marginal tentacles arrangements (Table 5). Labial and marginal directive tentacles are present in *C. brasiliensis* and absent in *C. ambonensis*. *Ceriantheomorphe brasiliensis* has only two pairs of mesenteries connected to the siphonoglyph (Fig. 4), while *C. adelita* sp. nov. and *C. ambonensis* have three. In *C. brasiliensis*, the directive mesenteries are longer than P3 and P5 unlike *C. adelita* sp. nov. and *C. ambonensis*. Both *C. brasiliensis* and *C. adelita* sp. nov. have directive mesenteries longer than betamesenteries (B and b), while in *C. ambonensis* the opposite happens (Table 5). Protomesenteries (P3) are longer than metamesenteries (m) in *C. adelita* sp. nov. unlike *C. ambonensis* and *C. brasiliensis*. All three species have distinct proportions between metamesenteries (M × m) and betamesenteries (B × b) disposed along the gastrovascular cavity and a distinct size relation between mesenteries directive and metamesenteries (m) (Table 5).

**Table 5.** Comparison of morphological characters between species of the genus *Ceriantheomorphe*.

Characters	*Ceriantheomorphe brasiliensis*	*Ceriantheomorphe adelita* sp. nov.	*Ceriantheomorphe ambonensis*
Number of marginal tentacles	132–392	192–352	48**-150*
Number of labial tentacles	108–384	144–336	72**-150*
Tentacular cycles	4	4	3*
Arrangement of marginal tentacles	(1)1123...	(?)1122...	(0)112...**
Arrangement of labial tentacles	(1)1122…	(?)1122…	(0)112…**
Proportion between pharynx in relation to body length	8–27%	21%	18%**
Siphonoglyph	Two pairs of mesenteries connected	Three pairs of mesenteries connected	Three pairs of mesenteries connected**
Proportion of gastrovascular cavity in relation to body length	33–72%	56%	55%**
Ratio between mesenteries	1.2–3.1% (B × b); 1.1–3.1%(m × M)	3% (B × b); 2.7–5.2% (m × M)	4% (B × b); 2.2–3.5% (M × m)**
P1 (directive mesenteries)	Longer than P3, P5, betamesenteries (B and b) and metamesenteries (m), except for m of the 2^nd^ and 3^rd^ cycles. Shorter than P2, P4 and metamesenteries (M).	Longer than betamesenteries (B and b) and metamesenteries (m). Shorter than P2, P3 and metamesenteries (M).	Shorter than mesenteries.**
P2	Longer than mesenteries	Longer than mesenteries	Longer than mesenteries
P3	Longer than P5, betamesenteries (b) and betamesenteries (B), except for B of the 1^st^ and 2^nd^ cycles. Shorter than directive mesenteries, P2, P4 and metamesenteries (M and m).	Longer than directive mesenteries, betamesenteries (B and b) and metamesenteries (m). Shorter than P2 and metamesenteries (M).	Longer than directive mesenteries and betamesenteries (B and b). Shorter than P2 and metamesenteries (M and m).
P4	Longer than directive mesenteries, P3, P5, betamesenteries (B and b) and metamesenteries (m), except for m of the 2^nd^ cycle. Shorter P2 and metamesenteries (M).	Absent	Absent
P5	Longer than betamesenteries (b) and betamesenteries (B), except for B from 1^st^ to 4^th^ cycles. Shorter than directive mesenteries, P2, P3, P4 and metamesenteries (M and m).	Absent	Absent
Proportion of directive mesenteries in the gastrovascular cavity	36.6%	30.5%	2.3%**
Proportion of protomesenteries P2 in the gastrovascular cavity	88.8%	92.5%	85.7%
Proportion of protomesenteries P3 in the gastrovascular cavity	12.2%	56.4%	14.2%
Proportion of protomesenteries P4 in the gastrovascular cavity	38.8%	Absent	Absent
Proportion of protomesenteries P5 in the gastrovascular cavity	11.1%	Absent	Absent

* Data from Kwietniewski (1898) ** Data from personal observation.

## Discussion

### Taxonomic studies

As a result of the disjunct distribution of specimens identified as *Ceriantheomorphe brasiliensis* (Mexico+US/Brazil+Uruguay) and the incomplete description of *Cerianthus ambonensis* made by Kwietniewski (1898) that later was proposed to be reassigned to the genus *Ceriantheomorphe* (Carlgren 1931), Den Hartog (1977) pointed out the need for a revision of Ceriantharia with special focus in the genus *Ceriantheomorphe*.

**Figure 5.**
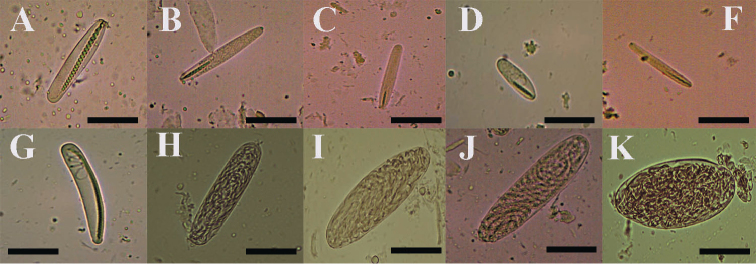
Cnidome of *Ceriantheomorphe brasiliensis*. **A** Microbasic b-mastigophore type I **B** microba-sic b-mastigophore type II **C** Microbasic b-mastigophore type III **D** microbasic b-mastigophore type IV **F** microbasic b-mastigophore type VI **G** microbasic b-mastigophore type V **H** ptychocyst type I **I** atrichous type I **J** holotrich **K** ptychocyst type II. Scale bars: 15 µm.

**Figure 6.**
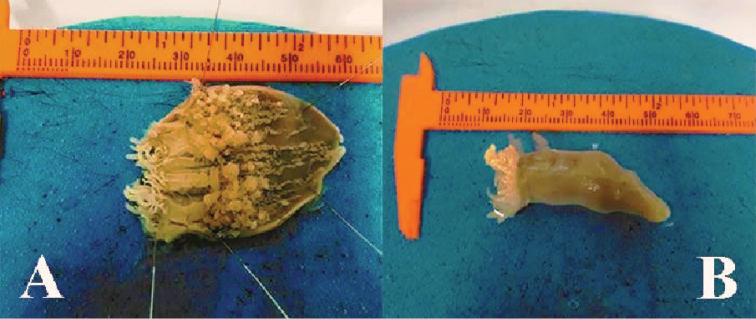
Specimen of *Ceriantheomorphe ambonensis* (MZSP 8476). **A** Dissected specimen and **B** external morphology.

Based on morphological characters and biogeographic perspectives (Table 5), we were able to identify two different morphotypes among specimens assigned as *Ceriantheomorphe brasiliensis*. Specimens from the Gulf of Mexico were recognized as an undescribed species, formally described here as *Ceriantheomorphe adelita* sp. nov. Some previous studies with *C. brasiliensis* from the South Atlantic have shown that this species has short-lived planula larvae (unpublished data). This trait could prevent long dispersion due to biogeographic barriers, and thus this species may not be capable of reaching the North Atlantic. This is a different pattern from that reported for Isarachnanthus nocturnus for *Isarachnanthus nocturnus*, which is able to disperse over long distances due to the presence of long-lived planktonic cerinula larvae (Stampar et al. 2012, 2015). Nonetheless, the maintenance of *C. brasiliensis* as a single species occurring in both northern and southern hemispheres would require some biogeographic events of which we have no evidence to date. Thus, in addition to the morphology, biogeographical understanding does not support the maintenance of these two populations as a single taxonomic unit.

**Figure 7.**
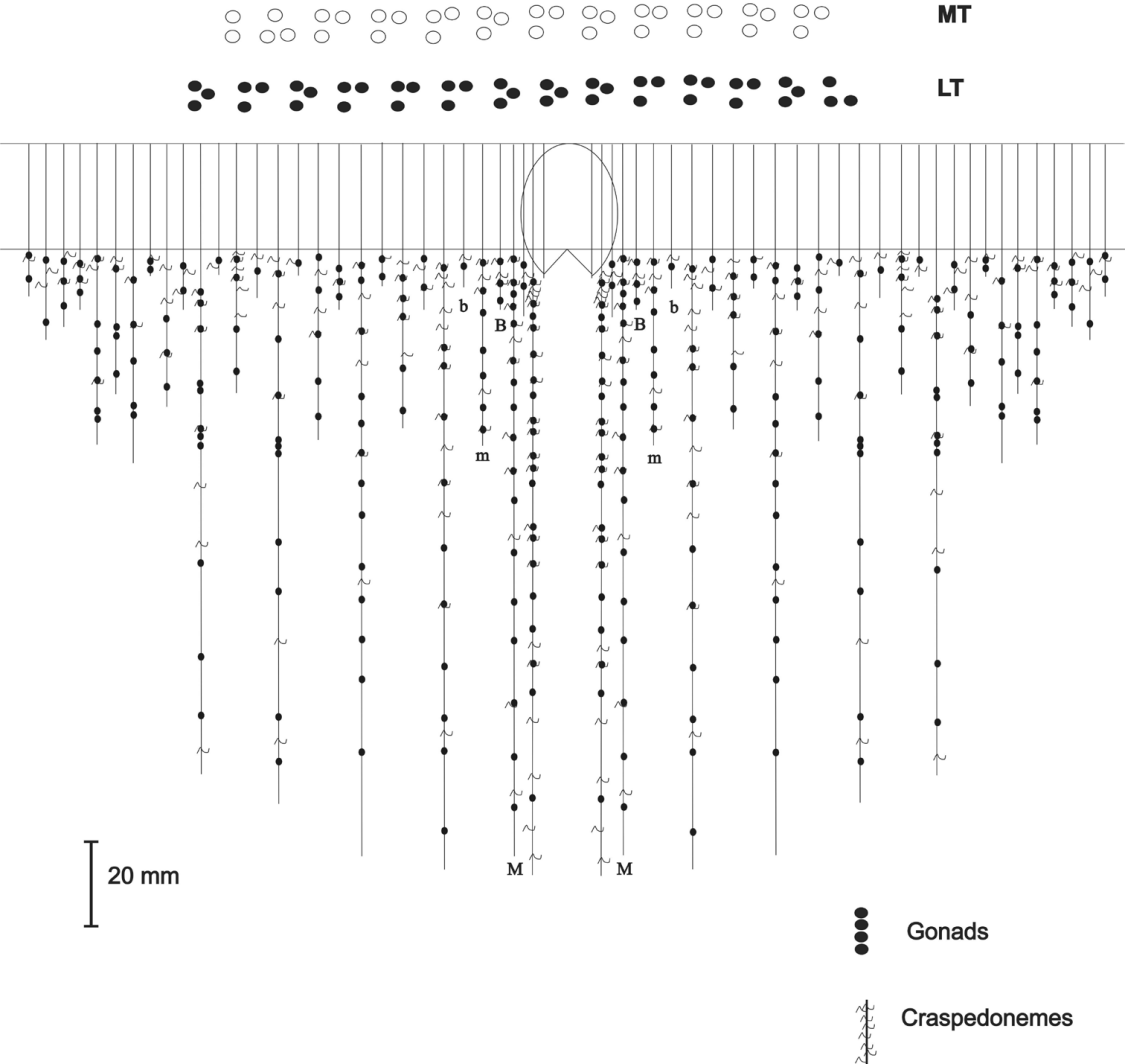
Mesenteries arrangement of *Ceriantheomorphe ambonensis*. **MT** Marginal tentacles **LT** Labial tentacles, **M** and **m**. Metamesenteries, **B** and **b**. Betamesenteries.

**Figure 8.**
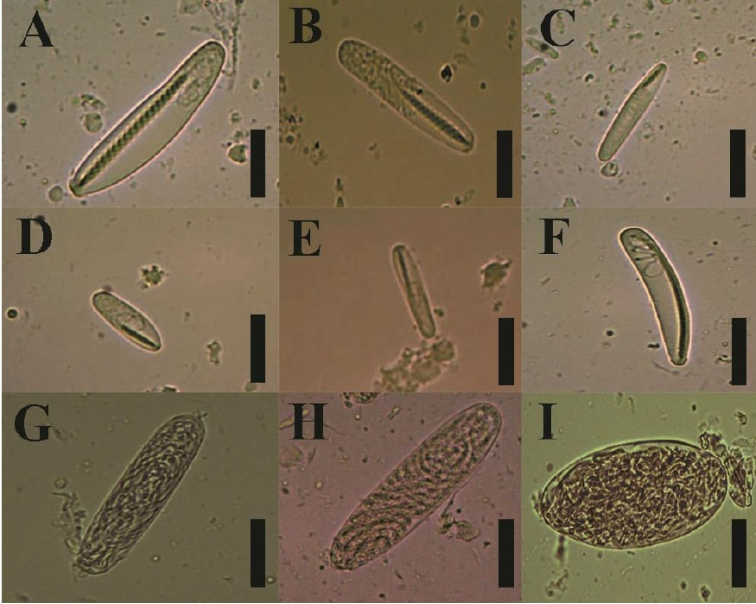
Cnidome of *Ceriantheomorphe ambonensis*. **A** Microbasic b-mastigophore type I **B** microbasic b-mastigophore type II **C** microbasic b-mastigophore type III **D** microbasic b-mastigophore type IV **E** microbasic b-mastigophore type V **F** microbasic b-mastigophore type VI **G** atrichous **H** holotrich **I** ptychocyst. Scale bars: 15 µm.

Carlgren and Hedgpeth (1952) argued that there were no differences between morphological characters in specimens from both areas (North and South Atlantic). We disagree with this assertion as *C. adelita* sp. nov. has several morphological characters that can distinguish it from other congeners. For instance, (1) marginal tentacles’ arrangement, ratio between metamesenteries (M × m) and betamesenteries (B × b), as well as the proportion occupied by protomesenteries (directive mesenteries, P2 and P3) in the gastrovascular cavity contrast with those observed in other *Ceriantheomorphe* (Table 5); (2) protomesenteries P3 are found connected to the siphonoglyph while the same is not observed in *C. brasiliensis*; (3) directive mesenteries are shorter than P3, unlike *C. brasiliensis*; (4) the number of mesenteries connected to the siphonoglyph is distinct in *C. brasiliensis* and *C. adelita* sp. nov.; (5) directive mesenteries are longer than betamesenteries (B and b), the same, however, is not observed in *C. ambonensis*; (6) directive mesenteries are longer than all metamesenteries (m), distinct from the other two species of the genus; (7) protomesenteries (P3) are longer than all betamesenteries (B), while in *C. brasiliensis* P3 are shorter than betamesenteries (B) of the 1^st^ and 2^nd^ cycles; (8) P3 are longer than metamesenteries (m), in contrast to those observed in *C. ambonensis* and *C. brasiliensis*; (9) protomesenteries (P4 and P5) are present in *C. brasiliensis*, while absent in the other species.

**Figure 9.**
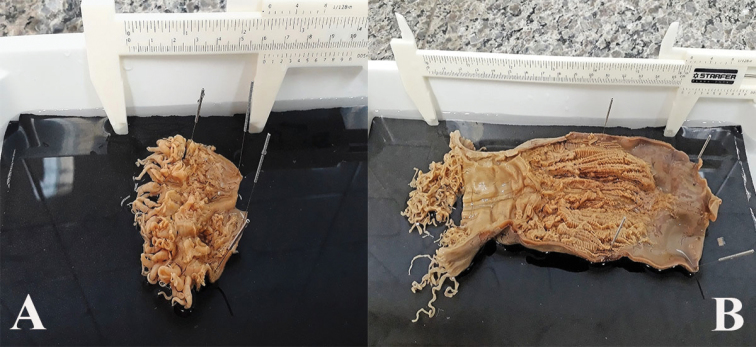
Specimens of *Ceriantheomorphe adelita* sp. nov. **A** Damaged specimen USNM 51253 from Louisiana **B** holotype specimen USNM 50015 from Corpus Christi (USA).

**Figure 10.**
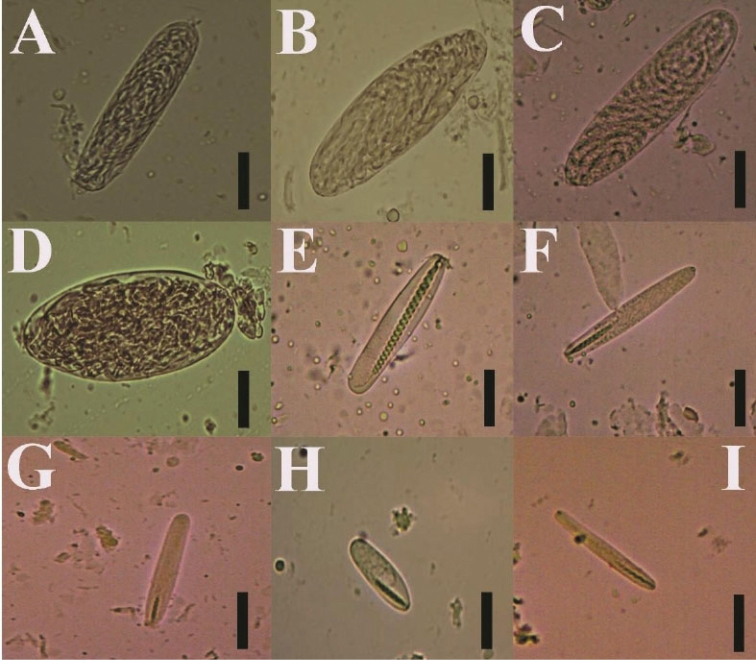
Cnidome of *Ceriantheomorphe adelita* sp. nov. holotype. **A** Atrichous **B** ptychocyst type I **C** holotrich **D** ptychocyst type II **E** microbasic b-mastigophore type I **F** microbasic b-mastigophore type II **G** microbasic b-mastigophore type III **H** microbasic b-mastigophore type IV **I** microbasic b-mastigophore type V. Scale bars: 15 µm.

Some authors have discussed the taxonomic value of mesenteriel organization regarding the assignment and identification of species (Carlgren 1912; Arai 1965; den Hartog 1977). Spier et al. (2012) have reported that *C. brasiliensis* in southern Brazil has two pairs of mesenteries connected to siphonoglyph. In this study, *C. adelita* sp. nov. was found to have three pairs. Nevertheless, our results also showed that the two species of *Ceriantheomorphe* from the Atlantic Ocean have different numbers of mesenteries connected to siphonoglyph, reinforcing the potential taxonomic value of this character.

Our results also demonstrated that the use of ratios (division of the values) between metamesenteries (M × m) and betamesenteries (B × b) for each quartet can be useful to distinguish species of *Ceriantheomorphe*. In specimens of *C. brasiliensis*, the ratio between metamesenteries (M × m) and betamesenteries (B × b) ranged from 1.1 to 3.1% and from 1.2 to 3.1%, respectively. In comparison, the ratios observed in *C. adelita* sp. nov. are from 2.7 to 5.2% between metamesenteries and 3% between betamesenteries, while in *C. ambonensis* they range from 2.2 to 3.5% between metamesenteries and 4% between betamesenteries.

Similar to the ratio between metamesenteries (M × m) and betamesenteries (B × b), the proportion of protomesenteries found in the gastrovascular cavity was also useful to distinguish *Ceriantheomorphe* species in our study. While protomesenteries (P3) in *C. adelita* sp. nov. extend over half of the entire gastrovascular cavity length (56.4%), the ones in *C. brasiliensis* and *C. ambonensis* are much shorter (12.2% and 14.2%, respectively). Furthermore, we found differences between species while comparing protomesenteries length (Table 5). In this way, we suggest that mesenteries have a taxonomic value when used comparatively.

**Figure 11.**
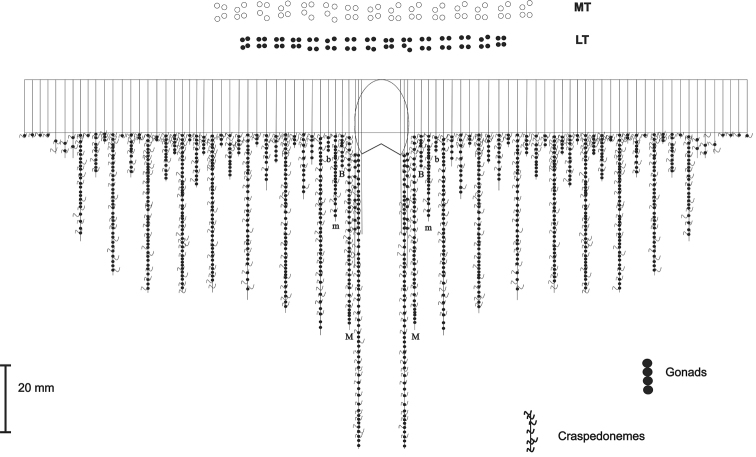
Mesenteries arrangement of *Ceriantheomorphe adelita* sp. nov. **MT** Marginal tentacles **LT** Labial tentacles **M** and **m** Metamesenteries **B** and **b** Betamesenteries.

**Table 6.** Compilation of morphological data on *Ceriantheomorphe ambonensis*.

Characters observed	Kwietniewski (1898)	This study
Specimen size	8.5 cm	3.8 cm
Number of marginal tentacles	About 150	24
Number of labial tentacles	About 150	36
Arrangement of both tentacles	3 cycles	3 cycles
Pharynx region	About 2.5 cm	0.7 cm long and 2.0 cm wide
Hyposulcus and hemisulci	No information	Absent
Gastrovascular cavity	Noinformation	2.1 cm long and 2.0 cm wide
Siphonoglyph	No information	0.7 cm long and 0.3 cm wide / 3 pairs of mesenteries connected.
Mesenteries	Numerous	96
Arrangement of mesenteries	No information	M,B,m,b
Cnidome	No information	Spyrocists, microbasic b-mastigophores (six types), atrichous (one type), ptychocyst and holotrichous.

### Geographic distribution of the genus *Ceriantheomorphe*

**Figure 12.**
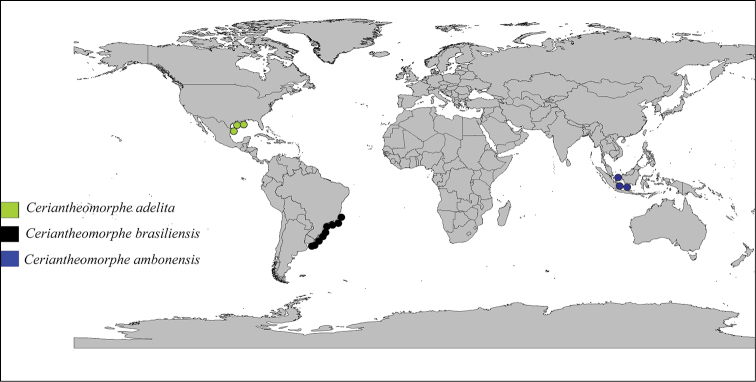
Distribution map of the genus *Ceriantheomorphe*.

Currently, the genus *Ceriantheomorphe* has a wide geographic distribution; one species is restricted to the warm temperate northwest Atlantic (Gulf of Mexico and United States of America), another to the warm temperate southwestern Atlantic (southeast and South of Brazil and Uruguay) and *C. ambonensis* is recorded from tropical Central Indo-Pacific, Sunda Shelf (Indonesia) (Spalding et al. 2007). However, there are no records of the genus *Ceriantheomorphe* from areas between the Atlantic and Indo-Pacific Oceans, which suggests that the genus has a disjunctive distribution, since there is no evidence of any connection between the extant populations of the valid species (Fig. 12).

Disjunctive distribution patterns are exhibited by some marine invertebrates, even those having a free-swimming phase that would benefit wide dispersal, for instance, the bivalve *Macoma balthica* Linnaeus, 1758 (Luttikhuizen et al. 2003) and the tunicate *Ciona intestinalis* Linnaeus, 1767 (Caputi et al. 2007). However, in the current case, in our opinion the disjunctive distribution of the genus *Ceriantheomorphe* is evidence of the need of further studies on the genus, especially focused on taxonomy in some under-investigated areas of the Indo-Pacific Ocean.

## Supplementary Material

XML Treatment for Ceriantheomorphe

XML Treatment for Ceriantheomorphe brasiliensis

XML Treatment for Ceriantheomorphe ambonensis

XML Treatment for Ceriantheomorphe adelita

## References

[B1] Arai MN (1965) A new species of *Pachycerianthus*, with a discussion of the genus and an appended glossary. Pacific Science 19(2): 205–218.

[B2] Caputi L Andreakis N Mastrototaro F Cirino P Vassillo M Sordino P (2007) Cryptic speciation in a model invertebrate chordate. Proceedings of the National Academy of Sciences of the United States of America 104(22): 9364–9369. 10.1073/pnas.0610158104 17517633 PMC1890500

[B3] Carlgren O (1912) Ceriantharia. The Danish Ingolf-Expedition 5(3): 1–78.

[B4] Carlgren O (1931) On some Ceriantharia. Arkiv för Zoology 23: 1–10.

[B5] Carlgren O (1940) A contribution to the knowledge of the structure and distribution of the cnidae in the Anthozoa. Kungliga Fysiografiska Sällskapets Handlingar 51: 1–62.

[B6] Carlgren O Hedgpeth JW (1952) Actiniaria, Zoantharia and Ceriantharia from shallow water in the northwestern Gulf of Mexico. Publications of the Institute of Marine Science University of Texas 2: 141–17.

[B7] den Hartog JC (1977) Descriptions of two new Ceriantharia from the Caribbean region, *Pachycerianthus curacaoensis* n. sp. and *Arachnanthus nocturnus* n. sp., with a discussion of the cnidom and of the classification of the Ceriantharia. Zoologische Mededelingen 51: 211–248.

[B8] Ehrenberg CG (1834) Beiträge zur physiologischen kenntnis der corallenthiereim allgemeinen, und besonders des rothen Meeres, nebsteinem versuche zur physiologischen systematik der selben. Abhandlungen der Königlichen Akademie der Wissenschaften, Berlin 1: 225–380.

[B9] Frey RW (1970) The Lebensspuren of some common marine invertebrates near Beaufort, North Carolina. II. Anemone burrows. Journal of Paleontology 44(2): 308–211. https://www.jstor.org/stable/1302545

[B10] González-Muñoz R Simões N Guerra-Castro EJ Hernández-Ortíz C Carrasquel G Mendez E Lira C Rada M Hernández I Pauls SM Croquer A Cruz-Motta JJ (2016) Sea anemones (Cnidaria: Actiniaria, Corallimorpharia, Ceriantharia, Zoanthidea) from marine shallow-water environments in Venezuela: new records and an updated inventory. Marine Biodiversity Records 9(18): 1–35. 10.1186/s41200-016-0016-7

[B11] Haswell WA (1883) Preliminary note on an Australian species of *Phoronis* (*Gephyrea tubicola*). Proceedings of the Linnean Society of New South Wales 7: 606–608. 10.5962/bhl.part.22767

[B12] Hedgpeth JW (1954) Anthozoa: the anemones. Fisheries Bulletin of the Fish and Wildlife Service (U.S.) 55: 285–290.

[B13] Kim IH Huys R (2012) Sabelliphilidae (Copepoda: Cyclopoida) associated with the tube anemone *Pachycerianthus maua* (Carlgren) and the horseshoe worm *Phoronis australis* Haswell off New Caledonia. Systematic Parasitology 83(1): 51–64. 10.1007/s11230-012-9369-4 22890380

[B14] Kwietniewski CR (1898) Actiniaria von Ambon und Thursday Island. In: Semon R (Ed.) Zoologische forschungsreisen in Australien und dem Malayischen archipel. Gustav Fischer Verlag, Jena 385–430.

[B15] Linnaeus C (1758) Systema Naturae per regna tria naturae, secundum classes, ordines, genera, species, cum characteribus, differentiis, synonymis, locis. Holmiae, 1–824. 10.5962/bhl.title.542

[B16] Linnaeus C (1767) Regnum Animale. In: Laurentii Salvii (Ed.) Systema naturae per regna tria naturae: secundum classes, ordines, genera, species, cum characteribus, differentiis, synonymis, locis. 1–532. 10.5962/bhl.title.157601

[B17] Luttikhuizen PC Drent J Baker AJ (2003) Disjunct distribution of highly diverged mitochondrial lineage clade and population subdivision in a marine bivalve with pelagic larval dispersal. Molecular Ecology 12: 2215–2229. 10.1046/j.1365-294X.2003.01872.x 12859640

[B18] Mariscal RN (1974) Nematocysts. In: Muscatine L, Lenhoff HM (Eds) Coelenterate biology-reviews and new perspectives. Academic Press PLACE, pp 129–178. 10.1016/B978-0-12-512150-7.50008-6

[B19] McMurrich JP (1910) Actiniaria of the Siboga expedition, Part I. Ceriantharia. Siboga-expeditie Monographes 10: 1–48.

[B20] Mello-Leitão CF (1919) *Cerianthus brasiliensis* – um novo cerianthoide americano. Archivos da Escola Superior de Agricultura e Medicina Veterinaria 3: 35–39.

[B21] Milne-Edwards H Haime J (1851) Archives du Muséum d’Histoire Naturelle. Archives du Muséum d’Histoire Naturelle 5: 1–504.

[B22] MMA Ministério do Meio Ambiente (2004) Lista de espécies de invertebrados aquáticos e peixes ameaçados de extinção. Instrução normativa n5, de maio de 2004. Diário Oficial da República Federativa do Brasil, Brasília, DF. Seção 1: 136–142.

[B23] Molodtsova TN (2009) Ceriantharia (Cnidaria) of the Gulf of Mexico. In: Felder DL Camp DK (Eds) Gulf of Mexico origin, waters, and biota: volume I, biodiversity.Texas A&M University Press, College Station, 365–367.

[B24] Nyholm KG (1943) Zur entwicklung und entwicklungs biologie der Ceriantharien und Aktinien. Zoologiska Bidrag från Uppsala 22: 87–248.

[B25] Pax F (1910) Studien an westindischen Aktinien. Zoologische jahrbucher. Abteilung für allgemeine Zoologie und Physiologie der Tiere, Suppl. 11: 157–330.

[B26] Perrier E (1893) Traité de zoologie: priméire partie – Zoologie génèrale protozoaires et phytózoaires arhtropodes. G. Masson, Paris, 1352 pp.

[B27] Rodriguez C Marques AC Stampar SN Morandini AC Christiansen E Genzano G Mianzan H (2011) The taxonomic position of the pelagic ‘staurozoan’ *Tesseragemmaria* as a ceriantharian larva. Zootaxa 2971: 49–58. 10.11646/zootaxa.2971.1.5

[B28] Silveira FL Morandini AC (2011) Checklist dos Cnidaria do Estado de São Paulo, Brasil. Biota Neotropica 11(1): 1–10. 10.1590/S1676-06032011000500016

[B29] Spalding MD Fox HE Allen GR Davidson N Ferdaña ZA Finlayson M Halpern BS Jorge MA Lombana AL Lourie SA Martin KD McManus E Molnar J Recchia CA Robertson J (2007) Marine ecoregions of the world: a bioregionalization of coastal and shelf areas. BioScience, 57(7): 573–583. 10.1641/B570707

[B30] Spier D Stampar SN Prantoni AL (2012) New record of the endangered cerianthid *Ceriantheomorphe brasiliensis* (Cnidaria: Hexacorallia) in Paranaguá Bay, southern Brazil. Marine Biodiversity Records 5(3): 1–4. 10.1017/S1755267212001078

[B31] Stampar SN Emig CC Morandini AC Kodja G Balboni A Silveira FL (2010) Is there any risk in a symbiotic species associating with an endengered one? A case of a phoronid worm growing on a case *Ceriantheomorphe* tube. Cahiers de Biologie Marine 51(2): 205–211.

[B32] Stampar SN Maronna MM Morandini AC Vermeij M Silveira FL (2012) Evolutionary diversification of banded-tube-dwelling anemones (Cnidaria; Ceriantharia; *Isarachnanthus*) in the Atlantic Ocean. PLoS ONE 7(7): e41091. 10.1371/journal.pone.0041091 PMC3397977 22815928

[B33] Stampar SN Maronna MM Kitahara MV Reimer JD Morandini AC (2014a) Fast-evolving mitochondrial DNA in Ceriantharia: a reflection of Hexacorallia paraphyly? PLoS ONE 9(1): e86612. 10.1371/journal.pone.0086612 PMC3903554 24475157

[B34] Stampar SN Morandini AC (2014) Occurrence of *Isarachnanthus* (Cnidaria: Anthozoa: Ceriantharia) at Ascension Island: a test of hypothesis. Journal of the Marine Biological Association of the United Kingdom 97(4): 1–5. 10.1017/S0025315414000423

[B35] Stampar SN Morandini AC Silveira FL (2014b) A new species of *Pachycerianthus* (Cnidaria, Anthozoa, Ceriantharia) from tropical southwestern Atlantic. Zootaxa 3827(3): 343–354. 10.11646/zootaxa.3827.3.4 25081164

[B36] Stampar SN Morandini AC Branco LC Silveira FL Migotto AE (2015) Drifting in the oceans: *Isarachnanthus nocturnus* (Cnidaria, Ceriantharia, Arachnactidae), an anthozoan with an extended planktonic stage. Marine Biology 162(11): 2161–2169. 10.1007/s00227-015-2747-0

[B37] Stampar SN Maronna MM Reimer JD Beneti JS Morandini AC (2016) Ceriantharia in current systematics: life cycles, morphology and genetics. In: Goffredo S Dubinsky Z (Eds) The Cnidaria, past, present and future: the world of medusa and her sisters.Springer International Publishing Switzerland, 61–72. 10.1007/978-3-319-31305-4_5

[B38] Tiffon Y (1987) Ordre des Cérianthaires. In: Grassé P (Ed.) Traité de zoologie: anatomie, systematique, biologie – Cnidaires / Anthozoaires.Masson, Paris, 211–257.

[B39] Verrill AE (1865) Classification of polyps (extract condensed from a synopsis of the polypi of the North Pacific exploring expedition, under captains Ringgold and Rodgers, U.S.N.). Proceedings of the Essex Institute 4: 145–152. 10.1080/00222936508679407

[B40] Vieira LM Stampar SN (2014) A new *Fenestrulina* (Bryozoa, Cheilostomata) commensal with tube-dwelling anemones (Cnidaria, Ceriantharia) in the tropical southwestern Atlantic. Zootaxa 3780(2): 365–374. 10.11646/zootaxa.3780.2.8 24871841

